# CHRNA9 as a New Prognostic Marker and Potential Therapeutic Target in Glioma

**DOI:** 10.7150/jca.92080

**Published:** 2024-02-24

**Authors:** Xiaoshan Ma, Ren Geng, Yao Zhao, Wanzhen Xu, Yao Li, Yining Jiang, Yuanhao Liu, Liyan Zhao, Yunqian Li

**Affiliations:** 1 Department of Neurosurgery, First Hospital of Jilin University, Changchun, China.; 2 Department of Neurosurgery, Qilu Hospital of Shandong University Dezhou Hospital, Dezhou, China.; 3 Department of Blood Transfusion, Second Hospital of Jilin University, Changchun, China.

**Keywords:** CHRNA9, Glioma, Prognostic Marker, STAT3 Pathway, Therapeutic Target

## Abstract

**Background:** The nicotinic acetylcholine receptor (nAChR) subunit alpha-9 (CHRNA9) is a unique cholinergic receptor, which is involved in tumor proliferation, apoptosis, metastasis and chemotherapy resistance. However, the correlation between the expression level of CHRNA9 in glioma and the clinical features and prognosis of glioma patients has not been clarified. The aim of this study was to verify the expression level of CHRNA9 in glioma and its effect on prognosis by bioinformatics methods.

**Methods:** The RNA-seq data of glioma and normal samples were obtained from the TCGA and GTEx databases. Bioinformatics methods were utilized to analyze the differential expression of CHRNA9 between tumor samples and normal samples. The potential association between CHRNA9 and the clinicopathological features of glioma patients was also investigated. The Kaplan-Meier method and Cox regression were utilized to analyze the relationship between CHRNA9 expression level and survival time and prognostic value of glioma patients. Enrichment analysis was applied to predict gene function and signaling pathways associated with CHRNA9. Experimental verification was performed using tumor tissues and paracancerous tissues from glioma patients.

**Results:** The results of bioinformatics analysis showed that the expression of CHRNA9 was increased in glioma tissues, correlating with poor prognosis and reduced patient survival time. Enrichment analysis suggested that CHRNA9 may interact with the JAK/STAT pathway. CHRNA9 was also found to be abnormally expressed in various other tumors and associated with the expression levels of numerous immune checkpoints in glioma. The findings from the analysis of clinical samples revealed that the expression levels of both mRNA and protein of CHRNA9 in glioma tissues were higher than those in paracancerous tissues. Similarly, the mRNA expression levels of STAT3, IL-6, and TNF-α, which are crucial factors in the STAT3 pathway, were elevated in glioma tissues compared to paracancerous tissues.

**Conclusion:** CHRNA9 is a potential prognostic marker and immunotherapy target for glioma, with its mechanism of action potentially linked to the STAT3 pathway.

## Introduction

Glioma is the most common malignant primary brain tumor in adults, accounting for about 30% of all primary brain tumors and 80% of all primary intracranial malignant tumors^1^-^3^. The morbidity of glioma is about 6 per 100,000 persons, with a male to female ratio of about 3:2^4^, ^5^. The risk factors for glioma mainly include ionizing radiation and some genetic syndromes^6^. Glioma has a high degree of malignancy and is difficult to treat, and its 5-year survival rate is less than 10%^3^. Gliomas typically originate from glial cells or precursor cells and develop into astrocytomas, oligodendrogliomas, ependymomas, or oligoastrocytomas^7^, ^8^. Gliomas exist in the brain in an aggressive manner and have an undefined boundary with normal brain tissue. The initial treatment of glioma usually involves surgical resection to maximize safety, so as to reduce tumor volume, and conduct accurate histological diagnosis and tumor genotyping through tissue analysis, followed by targeted radiotherapy (RT) and temozolomide (TMZ) chemotherapy^2^. It cannot be completely cured despite a combination of maximum surgical resection and standardized postoperative chemoradiotherapy^9^. Median survival of gliomas with active treatment after diagnosis is only about 2 years^1^, ^3^. In order to improve the survival quality of patients and alleviate the morbidity and mortality, incremental attention has been paid to adjuvant therapy other than surgery. Diagnosis and classification based on molecular biomarkers play a crucial role in the selection of glioma treatment, among which isocitrate dehydrogenase (IDH) status and 1p/19q codeletion have been confirmed to be related to the grade and prognosis of glioma^1^. An increasing number of biomarkers and their associated immunotherapies are being proposed and studied.

The nicotinic acetylcholine receptor (nAChR) is a receptor protein widely expressed on the cell membrane of human nerve tissue, and its clear function is to accept neurotransmitters and some ions to play the signal transduction between cells^10^. The nicotinic acetylcholine receptor (nAChR) subunit alpha-9 (CHRNA9) is a unique cholinergic receptor subtype that is mainly expressed in the cochlea and vestibular hair cells of the inner ear and is involved in the production of hearing^11^. Some studies have shown that CHRNA9 is expressed in immune cells, especially T cells^12^. CHRNA9 plays a role in exacerbating disease in inflammatory and autoimmune responses, while immune infiltration and T cell maturation are suppressed in CHRNA9 knockout mice^13^. CHRNA9 has been shown to be closely related to the occurrence and development of cancer. Nicotine can activate the STAT3 signaling pathway through CHRNA9 to upregulate the expression level of PD-L1 in cancer cells, and the transcriptional activator STAT3 regulates a variety of target oncogenes, affecting tumor proliferation, apoptosis, metastasis, and chemotherapy resistance^14^, ^15^. The expression level of CHRNA9 have been found to be elevated in malignant tumors and implicated in the advanced progression of colorectal cancer^16^. CHRNA9 has been shown to influence cell proliferation and transformation, promoting the onset and progression of lung cancer by regulating the cell cycle^17^, ^18^. The expression of CHRNA9 is specifically increased in breast cancer, and down-regulation of CHRNA9 expression can cause the growth cycle arrest of breast cancer cells, affect cell proliferation and migration, and inhibit the growth of cancer cells^19^. Although the significant roles of CHRNA9 in the disease progression of various tumors have been identified, its expression and mechanism of action in glioma remains unclear.

In our study, the expression of CHRNA9 and its primary mechanism of action in glioma were analyzed and predicted by bioinformatics methods. The analysis and prediction results were verified by collecting clinical glioma samples and para-cancerous samples. Our research hopes to further understand the disease pathogenesis of glioma, and provide new directions and theoretical basis for clinically targeted therapy of glioma.

## Methods

### Data source and preprocessed

All samples of RNA-sequencing (RNA-seq) data were downloaded from The Cancer Genome Atlas (TCGA) database (https://portal.gdc.cancer.gov/) and the Genotype-Tissue Expression (GTEx) database (https://www.gtexportal.org/). 516 glioma samples and 207 normal control samples were included in the study. The above sample data is preprocessed by University of California Santa Cruz (UCSC) XENA (https://xenabrowser.net/datapages/). The RNA-seq data was normalized to the format of transcripts per million (TPM) by the Toil process (a portable open-source workflow software, it can produce results faster and at a lower cost across diverse environments than such several other scientific workflow packages as Makeflow and Galaxy)^20^. After removing the missing values from the data, the samples were segmented into high and low expression groups according to the median of CHRNA9 gene expression level. The analysis of public data involved in this research complies with the data usage principles of TCGA and GTEx.

### Survival prognostic analysis

To explore the relationship between the expression of CHRNA9 in glioma patient samples and the prognosis of patients, the clinical survival data of all samples were extracted from the TCGA database. According to the median expression of CHRNA9 in glioma samples as the critical value, all glioma samples were divided into CHRNA9 high expression group and low expression group. Prognostic analysis of clinical samples was performed by R (version 3.6.3) software. Survminer R language package was used to analyze the effect of high and low expression of CHRNA9 on the overall survival (OS), disease-specific survival (DSS), and progression-free interval (PFI) of glioma patients. Combined with the World Health Organization (WHO) grade, 1p/19q codeletion, and IDH status of the samples, the relationship between CHRNA9 expression levels and OS in patients with different subtypes of glioma was analyzed. The prognostic analysis results were displayed using the Survminer R language package to draw Kaplan-Meier survival curves.

### Statistical analysis of clinical factors

The clinically relevant information on all glioma samples was downloaded from the TCGA database. COX regression was used to analyze the information about the primary therapy outcome, Age, WHO grade, and IDH status of the sample. Based on this result, a nomogram was built by RMS (6.2-0, https://cran.r-project.org/web/packages/rms/index.html) and survival (3.2-10, https://cran.r-project.org/web/packages/survival/index.html) online web tool. Finally, the boot method was used to repeat the calculation for 200 times, 40 samples in each group samples were verified against the nomogram, and a calibration curve was drawn.

### Gene differential expression analysis

Only the glioma sample data in the TCGA database were used, and the DESeq2 (1.26.0) R language package was used for gene expression difference analysis after preprocessing^21^. All glioma samples were divided into CHRNA9 high-expression group and low-expression group according to the median expression of CHRNA9, and differential analysis was performed to obtain differentially expressed genes (DEGs) between the two groups. The absolute value of Log2 fold change (FC) greater than 2 and the adjusted P value less than 0.01 were used as the screening criteria for DEGs. The results are presented in the form of a volcano graph.

### Single-cell datasets analysis of glioma cells

A single-cell transcriptome dataset of human glioma cells and immune cells completed by Abdelfattah et al in 2022 was analyzed using SingleCellPORTAL (https://singlecell.broadinstitute.org/single_cell).

### Pan-cancer analysis

TCGA project collected clinicopathologically annotated data along with multi-platform molecular profiles of more than 11,000 human tumors across 33 different cancer types^22^. RNA-seq data was collected from TCGA (https://portal.gdc.cancer.gov/) in UCSC Xena project (https://xenabrowser.net/datapages/). The UCSC XENA was used to analyze the mRNA expression levels of CHRNA9 in different types of tumor tissues and their paired normal tissues. At the same time, relevant normal sample data available in the GTEx (https://www.gtexportal.org/) database were integrated to supplement the above analysis. The expression of CHRNA9 gene in different tumors and corresponding normal tissues in the pretreated data was analyzed and a box diagram was drawn to compare the difference in expression between normal tissues and tumor tissues.

### Enrichment analysis

ClusterProfiler (3.14.3) R language package was used to perform Gene Ontology (GO) enrichment analysis and Kyoto encyclopedia of genes and genomes (KEGG) pathway enrichment analysis on the DEGs obtained by gene differential expression analysis^23^. The species was set as human, the adjusted P value less than 0.05 and q value less than 0.25 as the result screening criteria.

### Gene Set Enrichment Analysis (GSEA)

The gene set “C2.cp.v7.2.symbols.gmt” was downloaded from the MSigDB database (http://software.broadinstitute.org/gsea/msigdb) and used as a reference gene set. Based on the gene expression profiles of glioma patients in TCGA, GSEA analysis was performed using R software, and the adjusted P value was less than 0.05, and the q value was less than 0.25 as the result screening criteria. Enrichment pathways were ranked for each phenotype using P values and normalized enrichment scores (NES).

### Immune infiltration analysis

The single-sample Gene Set Enrichment Analysis (ssGSEA) method of Gene Set Variation Analysis (GSVA) module was used to analyze the correlation between the expression level of CHRNA9 and 24 types of immune cells. Spearman and Wilcoxon rank sum tests were used to analyze the degree of infiltration of immune cells in CHRNA9 gene high and low expression groups.

### Clinical Sample Collection

This experiment was approved by the Research Ethics Committee of the First Hospital of Jilin University (NO.19K127-002), and all patients gave informed consent. Tissue specimens were collected from the Neurosurgery Department of the First Hospital of Jilin University after resection of glioma lesions. 21 samples were stored in a -80°C freezer, including 14 gliomas samples and 7 para-cancer tissue samples. All methods were carried out in accordance with relevant guidelines and regulations.

### Real time-quantitative PCR (RT-qPCR)

Samples, each measuring 100 mg, were retrieved from the -80°C refrigerator and thawed on ice at 4°C. 1 ml Trizol reagent (15596026, Thermo Fisher Scientific, USA) was added to extract total RNA in the tissue. RNA was reverse transcribed into cDNA according to the reverse transcription kit (RT-01032, FOREGENE, Chengdu, China) as described. The primer sequences are shown in Table 1. Primers, cDNA, and SYBR green dye (11202ES03, Yeasen, Shanghai, China) were mixed at 20 μl and then amplified for 40 cycles to obtain the CT value of each well. The CT value of each sample was analyzed using the 2^-ΔΔCT^ method.

### Western blot

50 mg gliomas and para-cancer tissue samples were taken out from the -80°C refrigerator. RIPA lysate (P0013B, Beyotime Biotechnology, Shanghai, China) was added to lyse the tissue. The protein concentration of the sample was determined by the BCA method (Thermo Scientific MA, United States), and 10% SDS-PAGE gels (G2043, Servicebio, Wuhan, China) were configured according to the kit instructions for electrophoresis of the protein sample. After transferring to the polyvinylidene fluoride (PVDF) membrane (Merck Micropore, Burlington, MA, United States), incubate the corresponding antibody overnight according to the corresponding molecular weight. The relevant information about the antibody is shown in Table 2. After incubation with the secondary antibody corresponding to the primary antibody species for 1 h, the membrane was washed and visualized. At least 3 samples per group.

### Immunohistochemical analysis

The samples were fixed in 4% paraformaldehyde for 24 h and then decalcified. After embedding in paraffin, cut into 4 μm sections and place them on glass slides. Antigen retrieval was carried out after the sections were dewaxed and dehydrated, and the corresponding antibodies (Table 2) were incubated overnight in a 4°C refrigerator. After rinsing the slides with PBS, the corresponding secondary antibodies were incubated for color development. Representative images were collected under a light microscope at different magnifications.

### Statistical analysis

All data in this study were shown as means ± SD and statistically analyzed by SPSS 16.0 software. The independent sample t-test was used for comparison between the two groups, and P-value less than 0.05 was considered statistically significant.

## Results

### Differential expression of CHRNA9 in different clinical features

**Figure 1:**
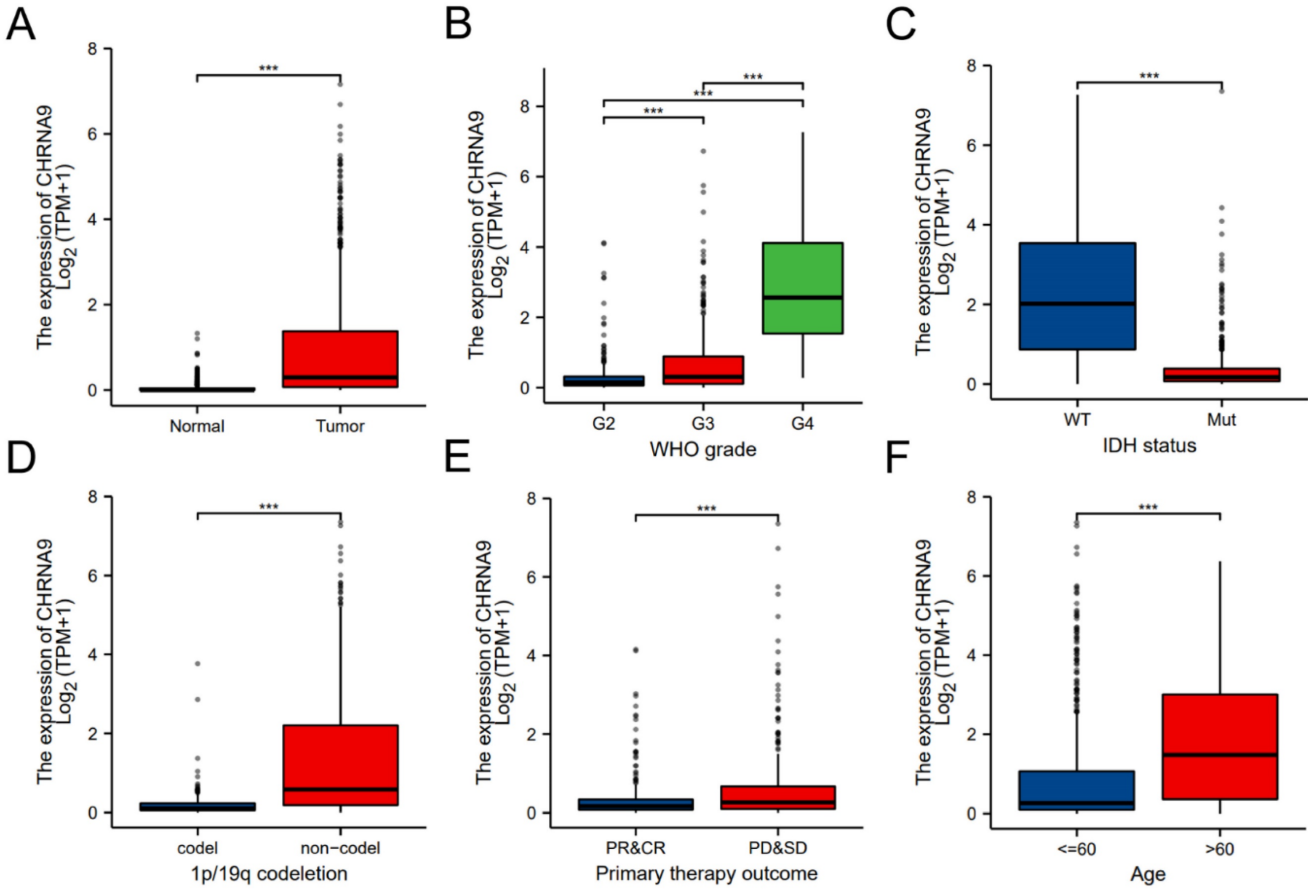
** Differential expression of CHRNA9 in different clinical features.** (A) Normal group and tumor group, (B) WHO grade, (C) IDH status, (D) 1p/19q codeletion, (E) Primary therapy outcome, (F) Age. ^***^P < 0.001

The expression level of CHRNA9 in the normal group and the glioma group in the TCGA database is shown in Figure 1A. Compared with the normal group, the expression level of CHRNA9 in the glioma samples was increased and the difference was statistically significant (*P* < 0.001). The expression of CHRNA9 in different WHO grades is shown in Figure 1B. Compared with grade 2, the expression level of CHRNA9 in grade 3 and grade 4 samples was significantly increased (*P* < 0.001), and the expression level of CHRNA9 in grade 4 samples was also higher than that in the grade 3 sample (*P* < 0.001). Compared with the IDH wild-type (WT) group, the expression level of CHRNA9 in the samples of the IDH mutant group was decreased (Figure 1C, *P* < 0.001). The expression level of CHRNA9 was also significantly increased in 1p/19q non-codeletion samples (Figure 1D, *P* < 0.001). The sample was divided into different groups based on the patient's outcome after primary therapy. Compared with the complete response-partial response (CR&PR) group, the expression level of CHRNA9 was increased in the stable disease-progressive disease (SD&PD) group of glioma samples (Figure 1E, *P* < 0.001). Age is also one of the factors related to the difference in the expression level of CHRNA9, the expression level of CHRNA9 in the Age > 60 group was significantly higher than that in Age ≤ 60 groups (Figure 1F, *P* < 0.001).

### CHRNA9 expression levels correlate with clinical features

The related information on the glioma sample's clinical features and gene expression was downloaded from the TCGA database. The correlation between clinical characteristics and CHRNA9 expression level was analyzed by the chi-square test and rank sum test. The analysis results showed (**Table 3**) that the expression level of CHRNA9 was correlated with WHO grade (*P* < 0.001), IDH status (*P* < 0.001), 1p/19q codeletion (*P* < 0.001), Primary therapy outcome (*P* < 0.001), Age (*P* < 0.001), and Age > 60 (*P* < 0.001).

### The expression level of CHRNA9 is correlated with the prognosis of glioma

The correlation between the expression level of CHRNA9 and the prognosis of glioma was analyzed by Cox regression (**Table 4**). The results of univariate Cox regression analysis showed that the high expression of CHRNA9 (*P* < 0.001), WHO grade (*P* < 0.001), IDH status (*P* < 0.001), Age (*P* < 0.001), Primary therapy outcome (*P* < 0.001), and 1p/19q codeletion (*P* < 0.001) were all associated with poor prognosis of glioma. Also, the results of multivariate Cox regression analysis showed that the high expression of CHRNA9 (*P* < 0.05) and clinical characteristics such as WHO grade (*P* < 0.01), IDH status (*P* < 0.001), Age (*P* < 0.001), and primary therapy outcome (*P* < 0.01) were all closely related to the poor prognosis of glioma patients.

### Patients with high expression of CHRNA9 have a shorter survival time

**Figure 2:**
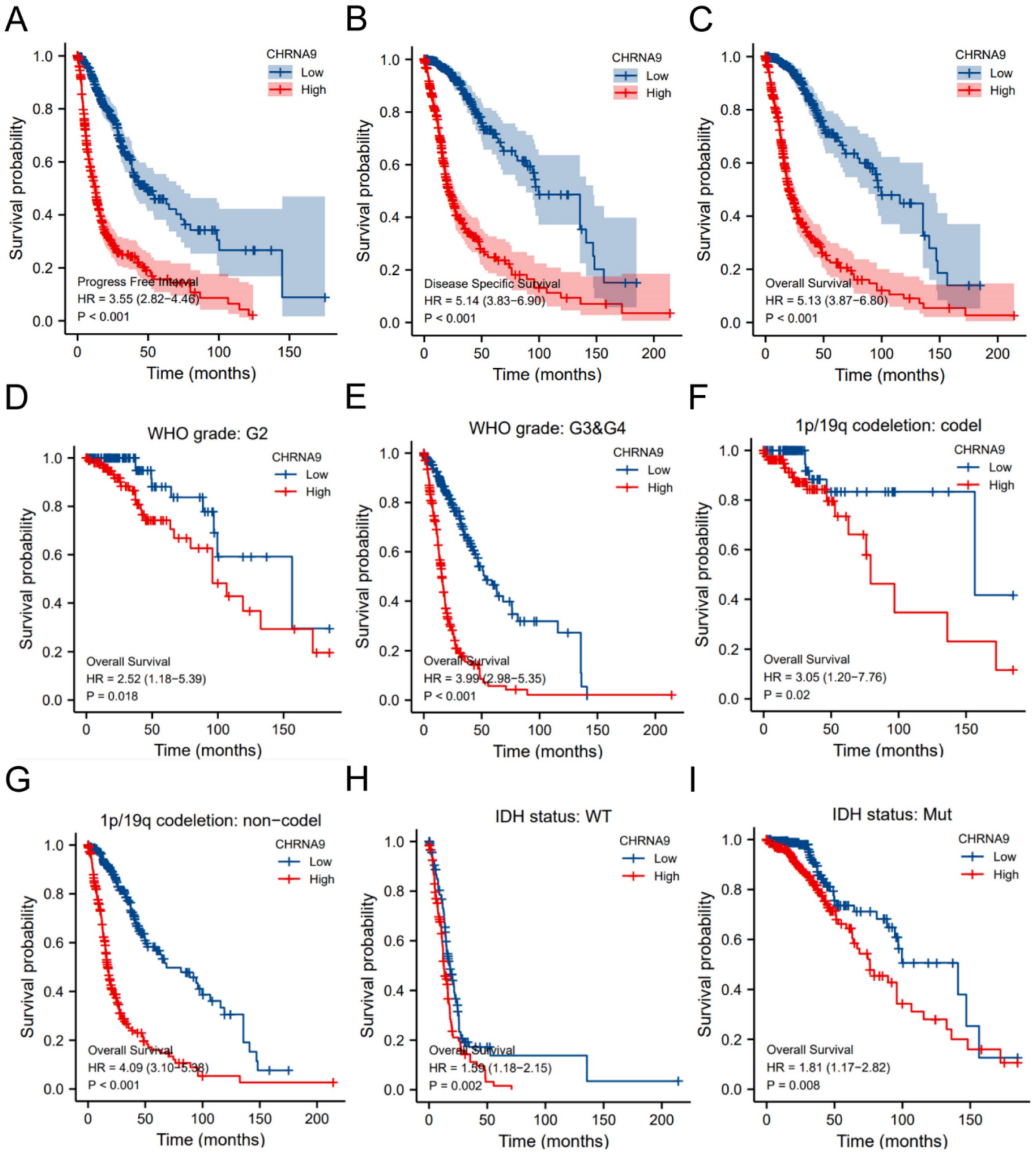
** High expression of CHRNA9 is not conducive to the prognosis of patients. (**A**)** PFI, (B) DSS, (C) OS, (D-E) WHO grade, (F) 1p/19q codeletion, (G) 1p/19q non-codeletion, (H) IDH wild-type, (I) IDH mutant.

Based on the median expression of CHRNA9 and the relevant clinical characteristics of glioma samples, glioma samples were divided into CHRNA9 high-expression and low-expression groups. The results of the survival analysis were shown in Figure 2. High expression of CHRNA9 was associated with shorter PFI (Figure 2A, *P* < 0.001), DSS (Figure 2B, *P* < 0.001), and OS (Figure 2C, *P* < 0.001). In WHO grade G2 (Figure 2D, *P* < 0.05) and G3&G4 (Figure 2E, *P* < 0.001) patients, high CHRNA9 expression was associated with shorter OS. In 1p/19q codeletion (Figure 2F) and non-codeletion (Figure 2G) samples, the OS of CHRNA9 high-expression samples was shorter (*P* < 0.05). Also, the OS of CHRNA9 high-expression samples was shorter (*P* < 0.01) in IDH WT (Figure 2H) and mutant (Figure 2I) samples.

### Foundation and verification of nomogram that correlated with CHRNA9

**Figure 3:**
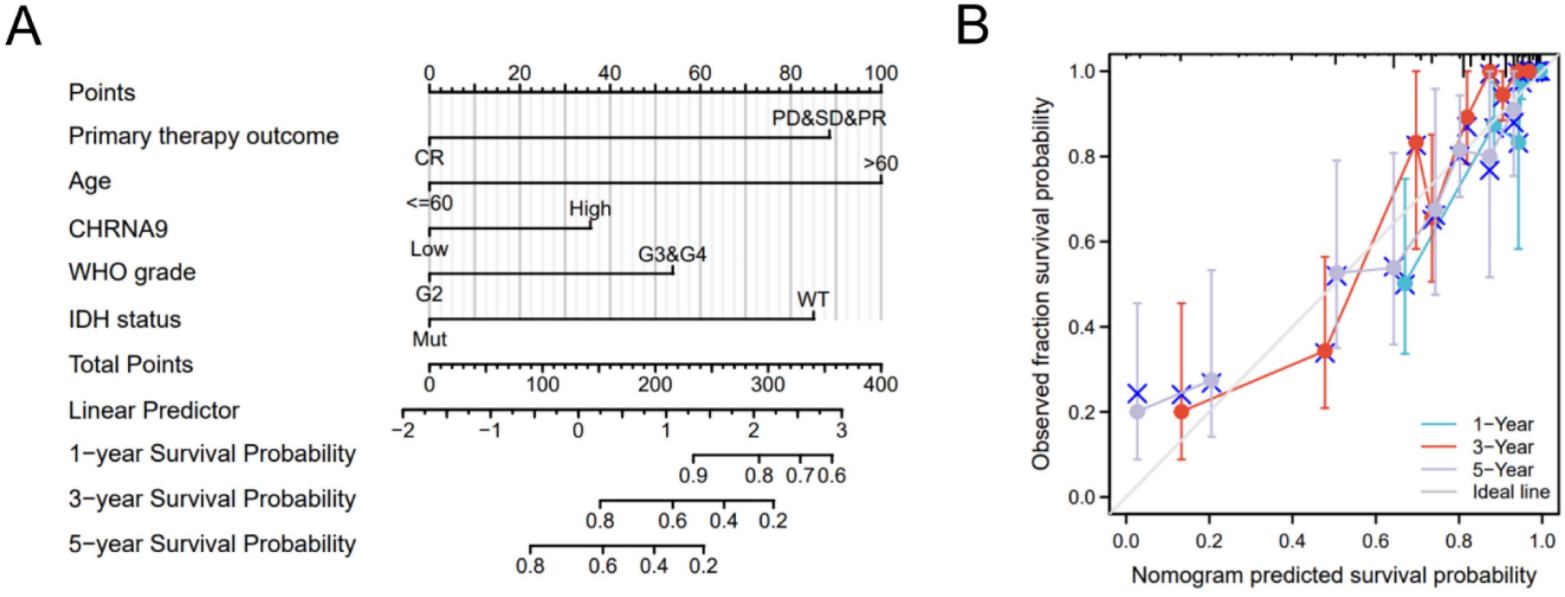
** Construction of CHRNA9-related prognostic nomogram.** (A) Nomogram survival prediction in patients with glioma. (B) The calibration curve displays the difference between the model prediction of 1-, 3-, and 5-year survival and actual survival outcomes.

The relationship between primary therapy outcome, Age, CHRNA9 expression level, WHO grade, IDH status, 1-year, 3-year, and 5-year survival prognosis of glioma was evaluated by nomogram [C-index: 0.849 (0.830-0.867), Figure 3A]. The calibration plot (Figure 3B) predicts the nomograms for 1-year, 3-year, and 5-year clinical outcomes, showing that the bias-corrected line is close to the ideal curve and the nomogram predictions are in good agreement with the actual results.

### Differential expression of CHRNA9 in pan-cancer

**Figure 4:**
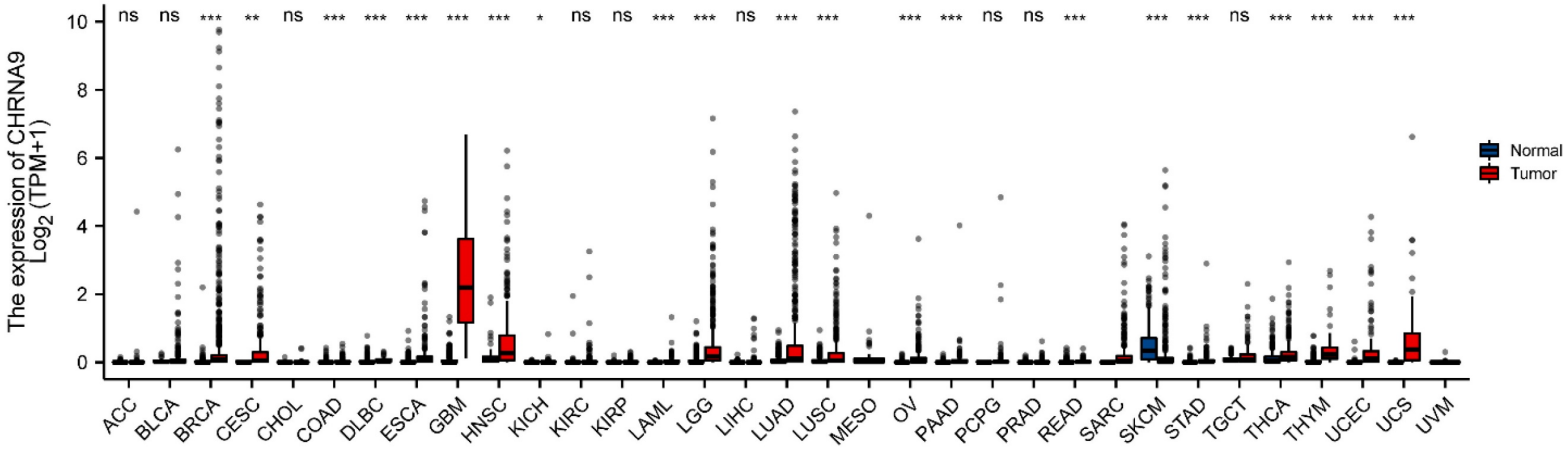
** CHRNA9 transcription levels in different tumor types.** ^*^P < 0.05, ^**^P < 0.01, ^***^P < 0.01 vs normal group.

The expression levels of CHRNA9 in different tumor types are shown in Figure 4. Compared with the normal group, the expression level of CHRNA9 in BRCA (*P* < 0.001), CESC (*P* < 0.01), COAD (*P* < 0.001), DLBC (*P* < 0.001), ESCA (*P* < 0.001), GBM (*P* < 0.001), HNSC (*P* < 0.001), KICH (*P* < 0.05), LAML (*P* < 0.001), LGG (*P* < 0.001), LUAD (*P* < 0.001), LUSC (*P* < 0.001), OV (*P* < 0.001), READ (*P* < 0.001), STAD (*P* < 0.001), THCA (*P* < 0.001), THYM (*P* < 0.001), UCEC (*P* < 0.001), and UCS (*P* < 0.001) were increased in diseased samples. But the expression level of CHRNA9 was lower than the normal group in SKCM (*P* < 0.001).

### Single-cell transcriptome analysis of human glioma and immune cells

**Figure 5:**
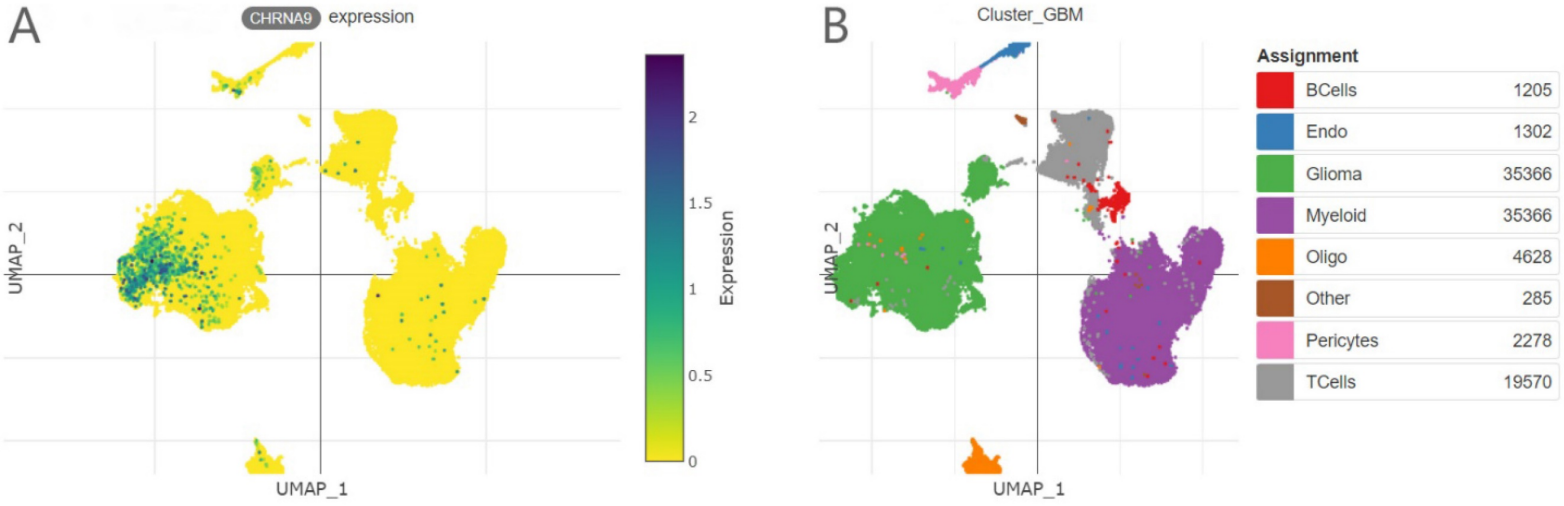
** The expression levels of CHRNA9 in different cell types.** (A) CHRNA9 expression levels. (B) the composition of different cell types glioma.

This single-cell UMAP prediction illustrates the composition of different cell types in human glioma (Figure 5B)^24^. According to the author's cell annotation criteria and results, single-cell sequencing showed that CHRNA9 was highly expressed in glioma cells, but relatively low in myeloid cells, T cells, and B cells (Figure 5A).

### CHRNA9 expression levels correlate with immune checkpoints

**Figure 6:**
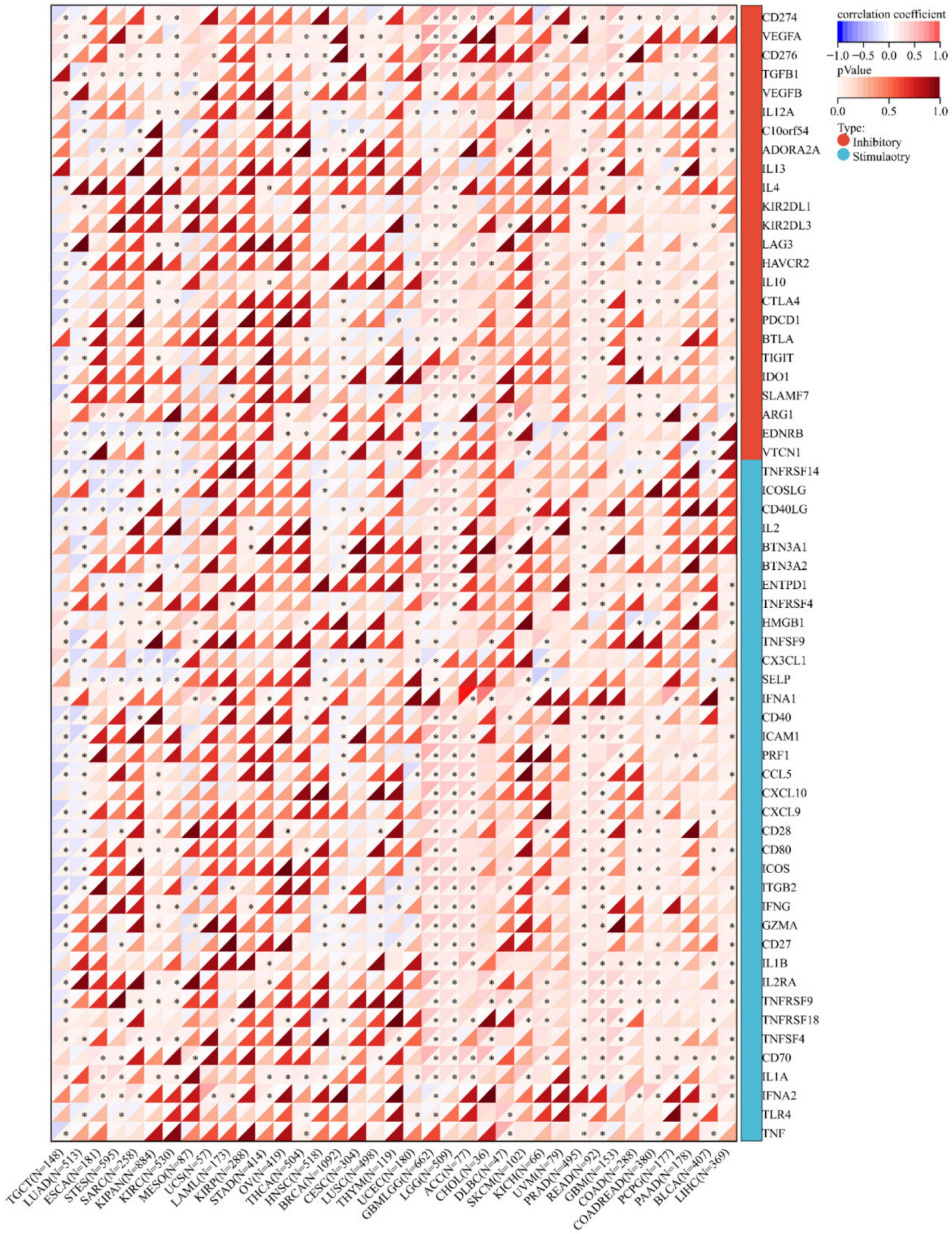
Correlation between CHRNA9 and immune checkpoints.

A total of 60 immune checkpoint genes were analyzed in this study, including 24 inhibitors and 36 stimulators. Pearson correlation analysis results (Figure 6) show that CHRNA9 positively correlated with immune checkpoint expression levels in most tumors. More importantly, the correlation between CHRNA9 and the expression levels of most immune checkpoints in glioma is statistically significant.

### 530 DEGs between CHRNA9 high expression and low expression group

**Figure 7:**
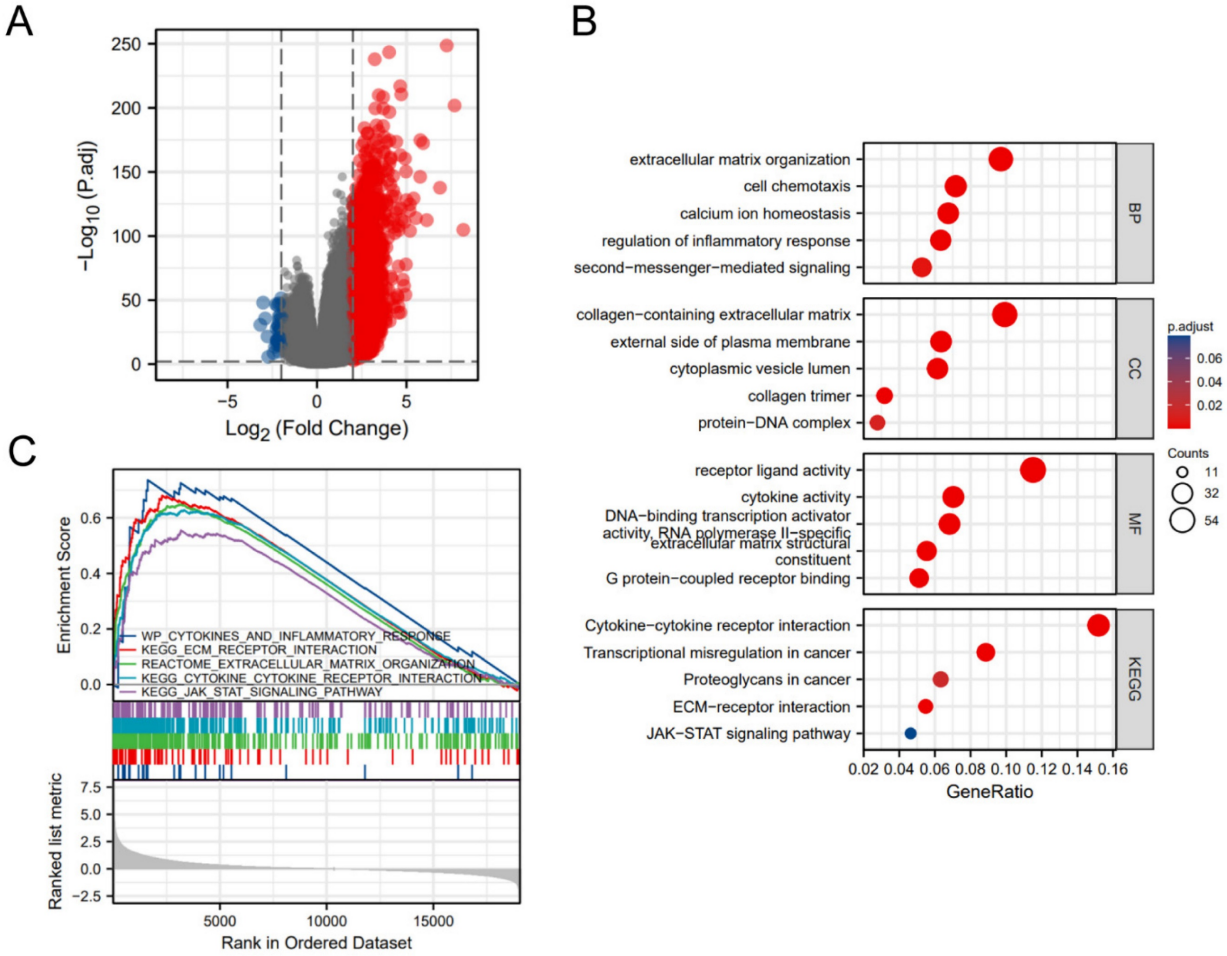
** Analysis of the role of CHRNA9 in glioma.** (A) DEGs between the high- and low- CHRNA9 expression groups. (B) The result of enrichment analysis. (C) The result of GSEA analysis.

Based on the median expression value of CHRNA9 in glioma samples in the TCGA database, they were divided into high expression group and low expression group, and the expression difference analysis results between the two groups were shown in the volcano plot (Figure 7A). A total of 530 DEGs were obtained by gene expression differential analysis, including 512 up-regulated genes (Log2 FC ≥ 2 and adjusted *P* value ≤ 0.01) and 18 down-regulated genes (Log2 FC ≤ -2 and adjusted *P* value ≤ 0.01).

### The functions of CHRNA9 in glioma

The results of the GO functional enrichment analysis are shown in Figure 7B. The top 5 terms of biological process (BP) include extracellular matrix organization (GO:0030198), cell chemotaxis (GO:0060326), calcium ion homeostasis (GO:0055074), regulation of inflammatory response (GO:0050727), and second-messenger-mediated signaling (GO:0019932). The top 5 terms of cellular component (CC) include collagen-containing extracellular matrix (GO:0062023), external side of plasma membrane (GO:0009897), cytoplasmic vesicle lumen (GO:0060205), collagen trimer (GO:0005581), and protein-DNA complex (GO:0032993). The top 5 terms of molecular function (MF) include receptor ligand activity (GO:0048018), cytokine activity (GO:0005125), DNA-binding transcription activator activity, RNA polymerase II-specific (GO:0001228), extracellular matrix structural constituent (GO:0005201), and G protein-coupled receptor binding (GO:0001664). KEGG pathway enrichment analysis results suggest that CHRNA9 may cooperate with cytokine-cytokine receptor interaction (hsa04060), transcriptional misregulation in cancer (hsa05202), proteoglycans in cancer (hsa05205), ECM-receptor interaction (hsa04512), and JAK-STAT signaling pathway (hsa04630) in glioma disease progression.

### CHRNA9 may interfere with the JAK-STAT pathway

The results of the GSEA enrichment analysis are shown in Figure 7C. This result suggests that CHRNA9 may participate in the progression of glioma disease by interfering with wp cytokines and inflammatory response (NES =1.80, *P* = 0.02), ECM receptor interaction (NES =1.74, *P* = 0.02), extracellular matrix organization (NES =1.71, *P* = 0.02), cytokine receptor interaction (NES =1.71, *P* = 0.02), and JAK STAT signaling pathway (NES =1.44, *P* = 0.04).

### CHRNA9 is specifically expressed in clinical glioma samples

**Figure 8:**
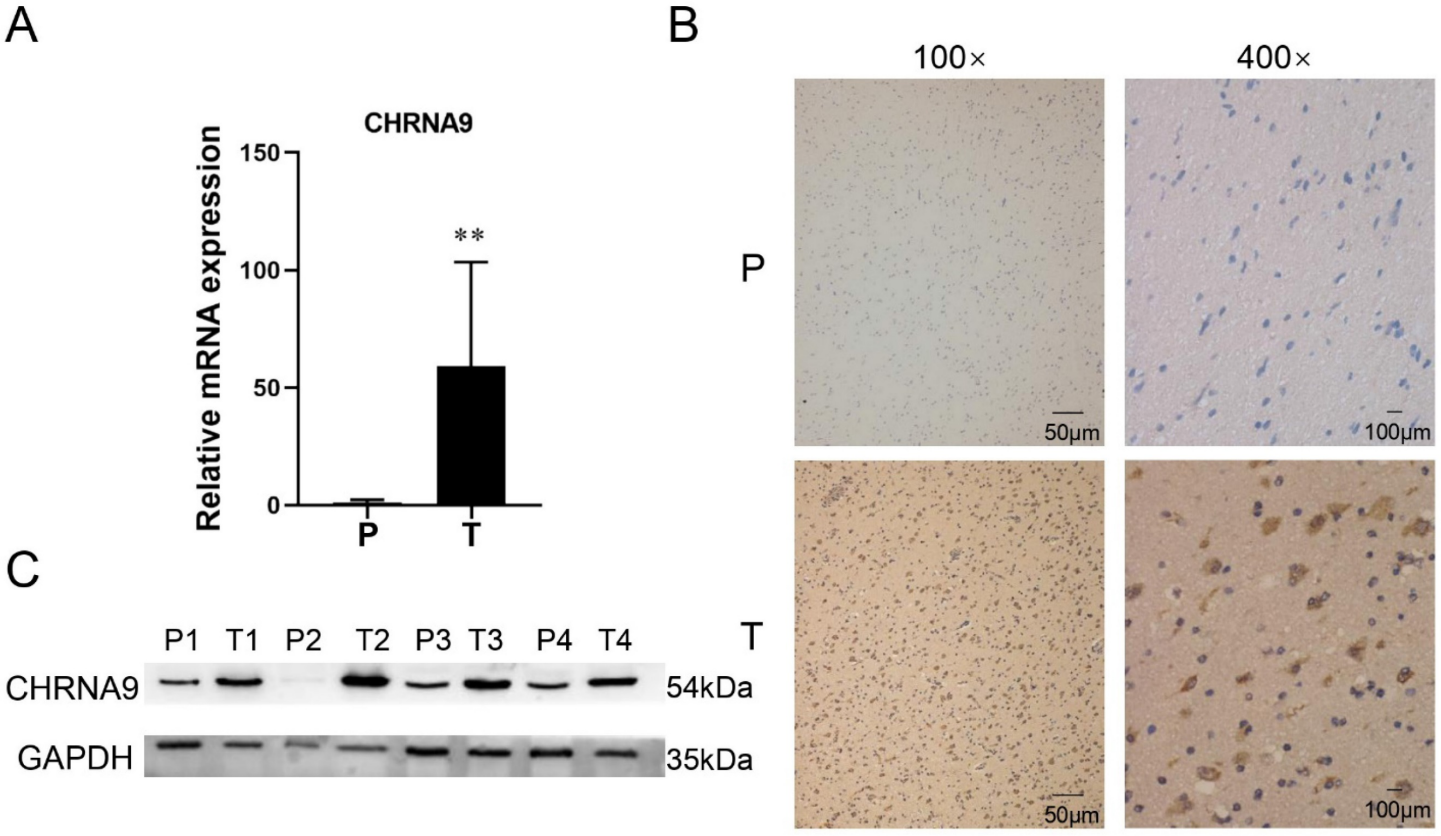
** CHRNA9 expression levels increased in glioma tissue samples.** (A) The mRNA expression level of CHRNA9 in glioma and para-cancer tissue was detected by RT-qPCR. The protein expression level of CHRNA9 in glioma and para-cancer tissue was detected by immunohistochemistry (B) and western blot (C). P, glioma para-cancer tissue; T, glioma tissue. ^**^P < 0.01 vs P group. Original blots are presented in Supplementary Figure 1 (GAPDH) and 2 (CHRNA9).

Clinical glioma and para-cancer tissue samples are used to detect the expression level of CHRNA9. The RT-qPCR results showed that compared with the para-cancer tissues, the mRNA expression level of CHRNA9 in the glioma samples was increased, and the difference was statistically significant (Figure 8A, *P* < 0.01). The representative picture of CHRNA9 protein expression in tumor samples detected by immunohistochemistry assay is shown in Figure 8B. The positive expression was brown-yellow granules in the cytoplasm, and the brown-yellow regions increased significantly in the tumor samples. The results of Western blot detection of the total CHRNA9 protein expression level in the samples showed (Figure 8C), compared with the para-cancerous samples, the CHRNA9 protein expression level in the tumor samples increased.

### Abnormal expression of STAT3 pathway in glioma tissue

**Figure 9:**
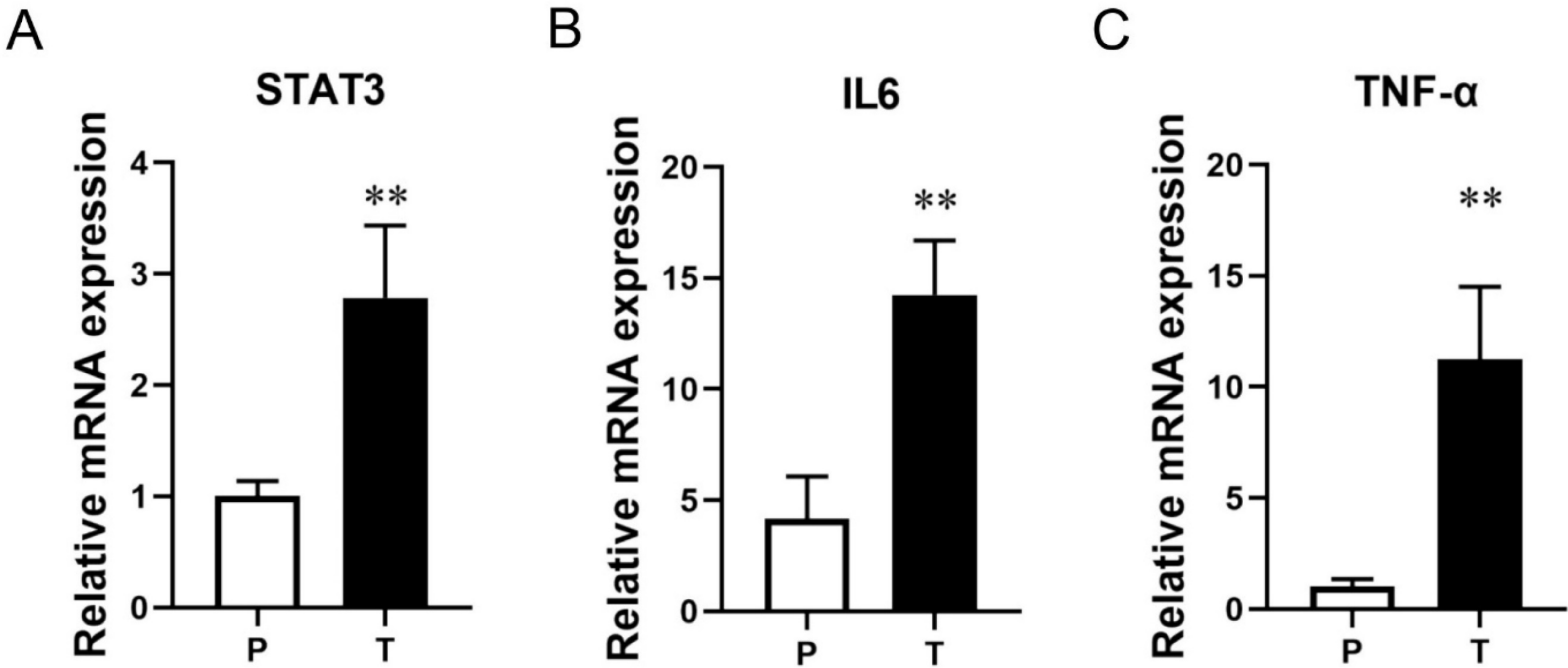
** The result of RT-qPCR assay.** The mRNA expression level of (A) STAT3, (B) IL-6, and (C) TNF-α in different groups. P, glioma para-cancer tissue; T, glioma tissue. ^**^P < 0.01 vs P group.

IL-6 and TNF-α play an important role in the STAT3 signaling pathway^25^. The mRNA expression levels of important factors in the STAT3 signaling pathway in glioma samples and paracancerous samples were detected by RT-qPCR assay. The results showed that the mRNA expression levels of STAT3 (Figure 9A, *P* < 0.01), IL-6 (Figure 9B, *P* < 0.01), and TNF-α (Figure 9C, *P* < 0.01) were significantly increased in glioma samples compared with paracancerous tissues.

### Relationship between CHRNA9 expression level and immune infiltration

**Figure 10:**
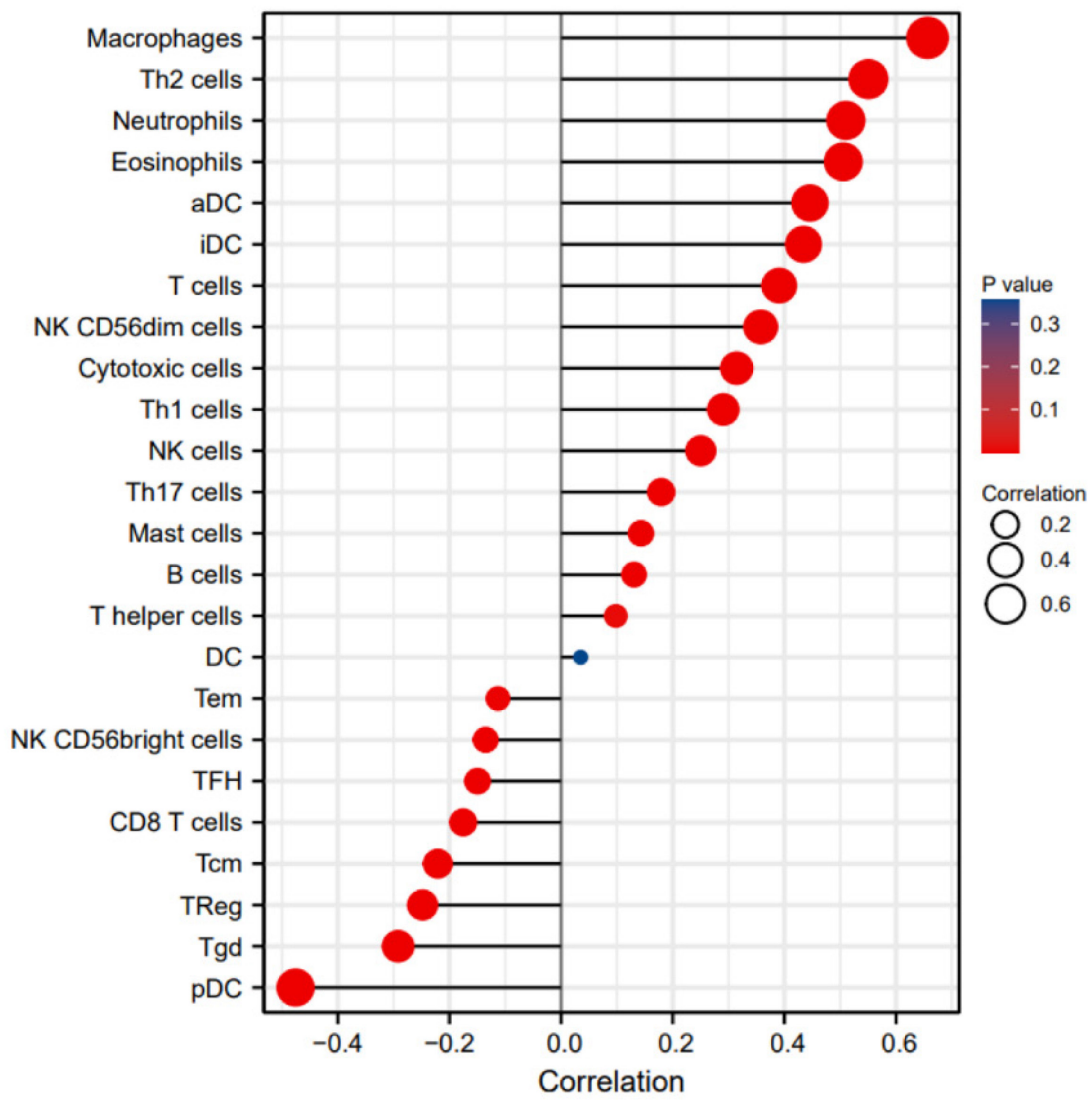
Association between abundance of immunocytes and CHRNA9 expression level.

The results of the correlation analysis between immune infiltration and the expression level of CHRNA9 are shown in Figure 10. The abundance levels of most immune cells in glioma tissues are correlated with the expression levels of CHRNA9 (*P* <0.05). Among them, Macrophages, Th2 cells, Neutrophils, Eosinophils, aDC, iDC, T cells, NK CD56dim cells, Cytotoxic cells, Th1 cells, NK cells, Th17 cells, Mast cells, B cells, T helper cells were positively correlated with the expression level of CHRNA9. Tem, NK CD56bright cells, TFH, CD8 T cells, Tcm, Treg, Tgd, and pDC were negatively correlated with the expression level of CHRNA9.

## Discussion

To date, gliomas are still notorious for its poor prognosis. The recurrence rate of radical surgical treatment of glioma is still exceedingly high, and its chemoradiotherapy is prone to produce drug resistance^9^. Even though many new therapies of glioma are currently being investigated, gliomas are difficult to be successfully implemented by clinical treatment due to its highly heterogeneous with multiple genetically distinct clones^26^. Therefore, elucidating the molecular mechanism of glioma tumorigenesis may provide theoretical foundation for developing effective therapeutic targets or seeking potential prognostic biomarkers. In this study, bioinformatics methods were used to analyze the clinically relevant information of glioma samples in the TCGA database, it was found that the expression level of CHRNA9 was correlated with the patients' WHO grade, IDH status, 1p/19q codeletion, Primary therapy outcome, and Age. We collected glioma and paracancerous samples from clinical patients and found that the mRNA expression level and protein expression level of CHRNA9 increased in glioma samples. The above aspects also have an impact on the prognosis of glioma patients. Survival analysis results manifested that the high expression of CHRNA9 was not conducive to the longer survival prognosis of patients. The result of enrichment analysis suggested that CHRNA9 may have a regulatory relationship with the JAK/STAT signaling pathway. CHRNA9 is also differentially expressed between tumor tissues and normal tissues in various cancer types and is associated with various immune cell abundance and immune checkpoints in glioma.

Immunotherapy is an emerging field of cancer treatment, and various immune checkpoint inhibitors have been found to perform well in the clinical treatment of various cancers^1^. More potential targeted therapeutic targets are still under investigation. The poor prognosis and high lethality of glioma are largely attributed to the highly invasive and migratory nature of glioma cells, which can migrate extensively and diffusely infiltrate into the surrounding brain tissue^27^. And mediating antiproliferative effects in malignant tumors may be an effective therapeutic strategy^28^. CHRNA9 has been shown to inhibit the proliferation of lung and breast cancer cells to alleviate disease progression^17^-^19^. In our study, the results of bioinformatics analysis by mining the transcriptome data of glioma samples in the TCGA database showed that the expression level of CHRNA9 was higher than that of the normal group.

Prognostic analysis results showed that WHO grade, IDH status, 1p/19q codeletion, Age, and primary therapy outcome were all associated with poor prognosis of glioma. The expression level of CHRNA9 was significantly increased in samples with high WHO grade, WT- IDH status, 1p/19q non-coding, Age > 60, and PD&SD. And the results of the survival analysis showed that the overall survival time of patients with high expression of CHRNA9 was lower than that of patients with low expression of CHRNA9. Similarly, the analysis of the expression levels of CHRNA9 in different types of cancer showed that the expression levels of CHRNA9 are increased in most types of cancer. In gliomas, CHRNA9 is correlated with expression levels at most immune checkpoints. These results suggest that high expression of CHRNA9 is not conducive to good prognosis and prolonged survival time of patients, and CHRNA9 is expected to become a new target for cancer immunotherapy. The above bioinformatics analysis results still need to be further verified by experiments. The mRNA expression level of CHRNA9 in glioma tissues was higher than that in paracancerous tissues found by RT-qPCR detection. Similarly, the protein expression level of CHRNA9 in glioma tissues was significantly higher than that in paracancerous tissues.

Although CHRNA9 has been shown to intervene in disease progression in lung and breast cancer, its mechanism of action in glioma disease progression has not been reported^17^, ^19^. In this study, the glioma samples in the TCGA database were divided into high expression group and low expression group according to the expression level of CHRNA9 by bioinformatics method. Pathway enrichment analysis based on DEGs between the two groups illustrated that there may be a regulatory relationship between CHRNA9 and the JAK/STAT signaling pathway during the progression of glioma disease, and CHRNA9 has been shown to activate STAT3^29^. The JAK/STAT signaling pathway is the core of extracellular cytokine-activated receptor-mediated signal transduction, which is mainly involved in cell proliferation, differentiation, and immune homeostasis^30^. The JAK/STAT signaling pathway is also involved in the progression, migration and invasion of glioblastoma^31^. The interaction of tumor cells, reactive astrocytes, and microglia in glioblastoma leads to high expression of TGF-β and IL-10, which promotes a positive feedback loop of STAT3 signaling and produces immune suppressive cytokine milieu^32^. Reactive astrocytes express IL-6 in the tumor microenvironment and increase STAT3 signaling through JAK activation^33^, ^34^. In glioblastoma, STAT3 has the most comprehensive oncogenic activity and immunosuppressive effects of all STAT family members^31^. Aberrant STAT3 signaling drives proliferation, neovascularization, apoptosis resistance, and immune evasion^35^. Activation of STAT3 signaling promotes self-renewal and tumorigenesis of glioblastoma stem cells^36^. STAT3 is also a key driver of diffuse invasion and glioma growth, so STAT3 may be an effective target for controlling glioma invasiveness^37^. And in our study, we found that the mRNA expression levels of IL-6, TNF-α, and STAT3, which are important factors in the STAT3 signaling pathway, were significantly increased in glioma samples. Targeting the JAK/STAT signaling pathway may help suppress the expression of target genes that control cell function and help treat cancer by causing cell death^31^, ^38^.

The JAK/STAT signaling pathway not only directly regulates the production of inflammatory factors, but also affects the function of immune cells^38^. Highly expressed STAT3 can inhibit the accumulation of effector T cells, thereby inhibiting their antitumor effects^39^. The signal transduction involved in STAT3 regulates the differentiation and immune-related functions of Th17 cells and myeloid-derived suppressor cells^25^, ^40^. In macrophages associated with infiltrating glioblastoma, STAT3 positively regulates the recruitment of associated macrophages and tumor growth^41^. The abundance and function of regulatory T (Treg) cells are also regulated by STAT3 signaling^42^. STAT3 can also inhibit the anti-tumor ability of dendritic cells by inhibiting the maturation, activation, and antigen presentation^25^. CHRNA9 is also expressed in a variety of immune cells, such as T cells, B cells, monocytes, and macrophages^43^. Inflammasome activation and IL-1β maturation in monocytes can be regulated by interfering with CHRNA9^44^. Although there is no relevant literature reporting the specific mechanism of CHRNA9 and immune cells, in our study we found that the expression level of CHRNA9 in glioma is correlated with the enrichment of most immune cells. Regulating the expression level of CHRNA9 may alleviate the level of immune infiltration in glioma tissue.

## Conclusion

Taken together, our study confirmed that CHRNA9 expression was increased in glioma, CHRNA9 overexpression was considered to be an independent factor for poor prognosis in glioma patients. CHRNA9 may intervene in the progression of glioma disease through the JAK/STAT pathway, and it may be a potential therapeutic target and prognostic biomarker for glioma.

## Supplementary Material

Supplementary figure.

## Figures and Tables

**Table 1:** The primer of genes

Gene	Primer	
CHRNA9	Forward	TGGACATATTCAACGCCTTGGACAG
	Reverse	TCAGAGCAGCAGCCATAGGAGATC
STAT3	Forward	CTTTGAGACCGAGGTGTATCACC
	Reverse	GGTCAGCATGTTGTACCACAGG
IL-6	Forward	TACCCCAGGAGAAGATTCC
	Reverse	TTTTCTGCCAGTGCCTCTTT
TNF-α	Forward	CTCTTCTGCCTGCTGCACTTTG
	Reverse	ATGGGCTACAGGCTTGTCACTC
β-actin	Forward	CGCCGCCAGCTCACCATG
	Reverse	CACATGCCGGAGCCGTTG

**Table 2:** The information of the primary antibody

Antibody	Dilution	cat	source
CHRNA9	1:500 for WB	26025-1-AP	Rabbit
CHRNA9	1:200 for IHC	26025-1-AP	Rabbit
GAPDH	1:2000 for WB	Ab8245	Mouse

**Table 3:** Relationship between CHRNA9 expression and clinic characteristics of glioma patients in TCGA

Characteristic	Low expression of CHRNA9	High expression of CHRNA9	*P*	statistic	method
n	348	348			
WHO grade, n (%)			< 0.001	225.77	Chisq.test
G2	173 (27.2%)	51 (8%)			
G3	126 (19.8%)	117 (18.4%)			
G4	2 (0.3%)	166 (26.1%)			
IDH status, n (%)			< 0.001	206.73	Chisq.test
WT	34 (5%)	212 (30.9%)			
Mut	314 (45.8%)	126 (18.4%)			
1p/19q codeletion, n (%)			< 0.001	98.04	Chisq.test
codel	143 (20.8%)	28 (4.1%)			
non-codel	205 (29.8%)	313 (45.4%)			
Primary therapy outcome, n (%)			< 0.001	26.43	Chisq.test
PD	52 (11.3%)	60 (13%)			
SD	100 (21.6%)	47 (10.2%)			
PR	48 (10.4%)	16 (3.5%)			
CR	104 (22.5%)	35 (7.6%)			
Gender, n (%)			0.193	1.7	Chisq.test
Female	158 (22.7%)	140 (20.1%)			
Male	190 (27.3%)	208 (29.9%)			
Race, n (%)			0.606	1	Chisq.test
Asian	5 (0.7%)	8 (1.2%)			
Black or African American	15 (2.2%)	18 (2.6%)			
White	321 (47%)	316 (46.3%)			
Age, n (%)			< 0.001	53.55	Chisq.test
<=60	316 (45.4%)	237 (34.1%)			
>60	32 (4.6%)	111 (15.9%)			
Age, meidan (IQR)	39 (31, 49.25)	53 (39, 63)	< 0.001	35828	Wilcoxon

**Table 4:** Cox regression analysis for clinical outcomes in glioma patients

Characteristics	Total (N)	Univariate analysis		Multivariate analysis	
		HR (95% CI)	*P* value	HR (95% CI)	*P* value
WHO grade	634				
G2	223	Reference			
G3&G4	411	5.642 (3.926-8.109)	<0.001	1.979 (1.256-3.119)	0.003
IDH status	685				
Mut	439	Reference			
WT	246	8.551 (6.558-11.150)	<0.001	2.585 (1.610-4.149)	<0.001
Age	695				
<=60	552	Reference			
>60	143	4.668 (3.598-6.056)	<0.001	3.658 (2.264-5.911)	<0.001
Primary therapy outcome	461				
CR	138	Reference			
PD&SD&PR	323	4.492 (2.187-9.226)	<0.001	3.152 (1.514-6.563)	0.002
CHRNA9	695				
Low	347	Reference			
High	348	5.128 (3.867-6.799)	<0.001	1.538 (1.030-2.295)	0.035
1p/19q codeletion	688				
codel	170	Reference			
non-codel	518	4.428 (2.885-6.799)	<0.001	1.511 (0.878-2.600)	0.136

## Abbreviations

nAChR: nicotinic acetylcholine receptor
CHRNA9: nicotinic acetylcholine receptor (nAChR) subunit alpha-9
TCGA: The Cancer Genome Atlas
GTEx: Genotype-Tissue Expression
UCSC: University of California Santa Cruz
TPM: transcripts per million
RT: radiotherapy
TMZ: temozolomide
IDH: isocitrate dehydrogenase
OS: overall survival
DSS: disease-specific survival
PFI: progression-free interval
WHO: World Health Organization
DEGs: differentially expressed genes
FC: Fold change
RNA-seq: RNA-sequencing
GO: Gene Ontology
KEGG: Kyoto encyclopedia of genes and genomes
GSEA: Gene Set Enrichment Analysis
NES: normalized enrichment scores
ssGSEA: single-sample Gene Set Enrichment Analysis
GSVA: Gene Set Variation Analysis
RT-qPCR: Real time-quantitative PCR
PVDF: polyvinylidene fluoride
WT: wild-type
CR: complete response
PR: partial response
SD: stable disease
PD: progressive disease
