# Supplementary figures and images for: CHRNA9 as a New Prognostic Marker and Potential Therapeutic Target in Glioma
Source: J Cancer. 2024 Feb 24;15(8):2095–109. doi: 10.7150/jca.92080 (PMC10937273; doi:10.7150/jca.92080)

# Supplementary Figures 1-2 (GAPDH, CHRNA9)

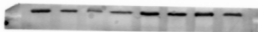

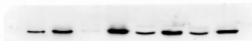

Supplement: Supplementary file 1 — Supplementary figure. [file jcav15p2095s1.pdf]
